# Outcomes to first-line pembrolizumab in patients with PD-L1-high (≥50%) non–small cell lung cancer and a poor performance status

**DOI:** 10.1136/jitc-2020-001007

**Published:** 2020-08-04

**Authors:** Joao V Alessi, Biagio Ricciuti, Elizabeth Jiménez-Aguilar, Fangxin Hong, Zihan Wei, Mizuki Nishino, Andrew J Plodkowski, Peter Sawan, Jia Luo, Hira Rizvi, Brett W Carter, John V Heymach, Mehmet Altan, Matthew Hellmann, Mark Awad

**Affiliations:** 1 Lowe Center for Thoracic Oncology, Dana Farber Cancer Institute, Boston, Massachusetts, USA; 2 Department of Medical Oncology, Hospital Universitario 12 de Octubre, Madrid, Comunidad de Madrid, Spain; 3 Department of Data Sciences, Dana Farber Cancer Institute, Boston, Massachusetts, USA; 4 Department of Radiology, Dana Farber Cancer Institute, Boston, Massachusetts, USA; 5 Department of Radiology, Memorial Sloan Kettering Cancer Center, New York, New York, USA; 6 Thoracic Oncology Service, Memorial Sloan Kettering Cancer Center, New York, New York, USA; 7 Druckenmiller Center for Lung Cancer Research, Memorial Sloan Kettering Cancer Center, New York, New York, USA; 8 Department of Thoracic/Head and Neck Medical Oncology, University of Texas MD Anderson Cancer Center, Houston, Texas, USA

**Keywords:** Lung Neoplasms, Tumor Biomarkers, Immunotherapy

## Abstract

**Background:**

Patients with non–small cell lung cancer (NSCLC) and a poor Eastern Cooperative Oncology Group Performance Status (ECOG PS) have been excluded from phase III immunotherapy clinical trials. We sought to evaluate clinical outcomes to first-line pembrolizumab in patients with advanced NSCLC, a PD-L1 Tumor Proportion Score (TPS) of ≥50%, and an ECOG PS of 2.

**Methods:**

We performed a multicenter retrospective analysis of patients with metastatic NSCLC and a PD-L1 TPS of ≥50% (negative for genomic alterations in *EGFR* and *ALK*) who received treatment with first-line pembrolizumab. Clinical outcomes were compared in patients based on ECOG PS.

**Results:**

Among the 234 patients, 83.3% (n=195) had an ECOG PS of 0 or 1, and 16.7% (n=39) had an ECOG PS of 2. The baseline clinicopathological characteristics were balanced between the ECOG PS 0–1 vs 2 groups in terms of age, sex, tobacco use, histology, *KRAS* mutation status, presence of other potentially targetable driver mutations (*BRAF, MET, HER2, RET*), presence of brain metastases, and PD-L1 TPS distribution. Compared with patients with an ECOG PS of 0 or 1, patients with an ECOG PS of 2 had a significantly lower objective response rate (43.1% vs 25.6%; p=0.04), a numerically shorter median progression-free survival (6.6 months vs 4.0 months; HR 0.70 (95% CI 0.47 to 1.06); p=0.09), and a significantly shorter median overall survival (20.3 months vs 7.4 months; HR 0.42 (95% CI 0.26 to 0.68); p<0.001). On disease progression, patients with an ECOG PS of 2 were significantly less likely to receive second-line systemic therapy compared with patients with an ECOG PS of 0–1 (65% vs 22.2%, p=0.001).

**Conclusions:**

A subset of patients with NSCLC and an ECOG PS of 2 can respond to first-line pembrolizumab. However, clinical outcomes in this population are often poor and use of second-line systemic therapy is infrequent.

## Background

The use of programmed cell death-1 (PD-1) pathway immune checkpoint inhibitors (ICIs) has become the standard of care for the initial treatment of advanced non–small cell lung cancer (NSCLC). Thus far, published phase III NSCLC clinical trials with PD-1 pathway inhibitors in the first-line setting have been restricted to patients with a good Eastern Cooperative Oncology Group Performance Status (ECOG PS) of 0 or 1.^1–4^ However, an estimated 21% of patients with advanced NSCLC have an ECOG PS of 2 at the time of initial diagnosis,^5^ and little is known about the efficacy of first-line immunotherapy in this population.

Prospective trials for NSCLC have only recently begun to report outcomes for patients with a poor ECOG PS who received prior platinum-based chemotherapy followed by immunotherapy. Of the 1426 previously treated patients who subsequently received nivolumab monotherapy in the CheckMate 153 NSCLC trial, the median overall survival (mOS) for the 128 (9%) patients with an ECOG PS of 2 was 4.0 months, compared with 9.1 months for the overall population of patients with an ECOG PS 0-2.^6^ PD-L1 levels were not available in the majority of patients on this study, and only 12 patients were known to have a PD-L1 Tumor Proportion Score (TPS) of ≥50%. Similar results were reported in the CheckMate 171 study of nivolumab in previously treated squamous NSCLC, where the ECOG PS 2 population had an mOS of 5.2 months.^7^

Since there is a paucity of data on the efficacy of immunotherapy in treatment-naïve patients with NSCLC, a PD-L1 TPS of ≥50%, and an ECOG PS of 2, we sought to assess clinical outcomes to first-line pembrolizumab in this population.

## Methods

Patients in this multicenter retrospective analysis were included if they had *EGFR*/*ALK*-negative advanced NSCLC with a PD-L1 TPS of ≥50% and received at least one dose of first-line commercial pembrolizumab monotherapy outside the setting of a clinical trial and who had an ECOG PS documented at the start of immunotherapy treatment. Participating academic medical centers included the Dana-Farber Cancer Institute, the Memorial Sloan Kettering Cancer Center, and the MD Anderson Cancer Center. Patients were included if they had consented to institutional review board–approved medical record review protocols at each institution.

The objective response rate (ORR) and progression-free survival (PFS) were determined by blinded radiology review using Response Evaluation Criteria In Solid Tumors (RECIST) version 1.1. PFS was defined as the time from pembrolizumab start to progression or death, and for those without progression, censoring was done at the time of the last disease assessment scan showing no progression. Overall survival (OS) was calculated from pembrolizumab start to death. Patients who were still alive at the time of data analysis were censored at the date of last contact. Event-time distributions were estimated using Kaplan-Meier methodology. Log-rank tests were used to test for differences in event-time distributions, and Cox proportional hazards models were used to estimate HRs in univariate and multivariate models for PFS and OS. Variables demonstrating signal of association with p<0.2 in univariate analysis were included in the multivariate model; a purposeful selection method was used with the consideration of possible two-way interactions.^8^ χ^2^ test was used to compare proportions. All p values are two sided and CIs are at the 95% level, with significance predefined to be at the two-sided 0.05 level.

## Results

### Baseline patient clinical factors by ECOG PS groups

Of the 234 patients who received at least one dose of commercial pembrolizumab in the first-line setting for NSCLC with a PD-L1 TPS of ≥50%, 195 (83.3%) had an ECOG PS of 0–1 and 39 (16.7%) had an ECOG PS of 2 at the start of immunotherapy treatment. The baseline clinicopathological characteristics were generally balanced between the two cohorts in terms of age, gender, smoking status, histology, *KRAS* mutation status, presence of other potentially targetable oncogenic driver mutations (*BRAF*, *MET*, *HER2*, *RET*), history of brain metastases, and PD-L1 TPS distribution (table 1).

**Table 1:** Distribution of clinical characteristics by ECOG PS

Clinical characteristic	ECOG PS 0–1	ECOG PS 2
	N=195 (%)	N=39 (%)
Age, median (range)	68 (35-92)	73 (43-91)
Age		
<70	111 (56.9)	17 (43.6)
≥70	84 (43.1)	22 (56.4)
Sex		
Male	95 (48.8)	20 (51.3)
Female	100 (51.2)	19 (48.7)
Smoking status		
Current/former	177 (90.8)	37 (94.9)
Never	18 (9.2)	2 (5.1)
Histology		
Adenocarcinoma	150 (76.9)	30 (76.9)
Squamous cell carcinoma	25 (12.8)	6 (15.4)
NSCLC NOS	20 (10.3)	3 (7.7)
Oncogenic driver mutation		
KRAS	65 (37.6)	16 (45.7)
Potentially targetable oncogenes*	26 (15.0)	3 (8.6)
None identified	82 (47.4)	16 (45.7)
N.A.	22	4
Brain metastases		
Absent	146 (74.9)	24 (61.5)
Present	49 (25.1)	15 (38.5)
PD-L1 expression		
≥90%	99 (50.8)	17 (43.6)
50%–89%	96 (49.2)	22 (56.4)

*Including (*BRAF*, *MET*, *HER2*, *RET*).

ECOG PS, Eastern Cooperative Oncology Performance Status; N.A., not available; NSCLC NOS, non–small cell lung cancer not otherwise specified; PD-L1, programmed cell death ligand 1.

### Clinical outcomes

**Figure 1:**
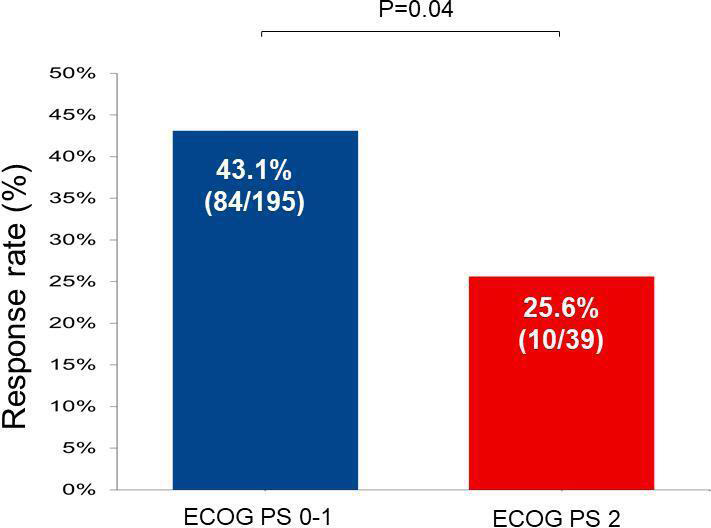
Response rate to first-line pembrolizumab in the Eastern Cooperative Oncology Performance Status (ECOG PS) 0-1 and ECOG PS 2 groups.

**Figure 2:**
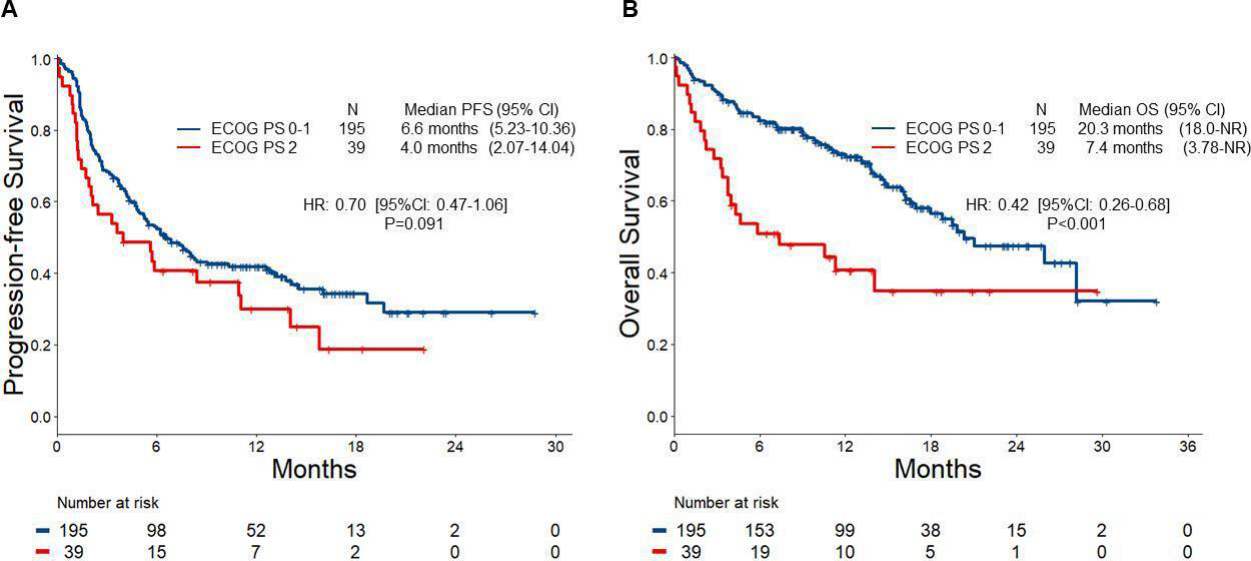
Kaplan-Meier curves for (A) progression-free survival (PFS) and (B) for overall survival (OS) to first-line pembrolizumab in the Eastern Cooperative Oncology Group Performance Status (ECOG PS) 0-1 and ECOG PS 2 groups.

In the entire cohort of 234 patients treated with first-line commercial pembrolizumab, the ORR was 40.2% (95% CI 33.8% to 46.8%). At a median follow-up of 14.8 months (95% CI 13.5 to 16.7), the mPFS was 6.2 months (95% CI 4.9 to 8.4), and the mOS was 19.8 months (95% CI 16.2, not reached). Compared with patients with an ECOG PS of 0 or 1, patients with an ECOG PS of 2 had a significantly lower objective response rate (ORR 43.1% vs 25.6%; p=0.04, figure 1), a numerically shorter median progression-free survival (median PFS 6.6 months vs 4.0 months; HR 0.70 (95% CI 0.47 to 1.06); p=0.09, figure 2A), and a significantly shorter overall survival (mOS 20.3 months vs 7.4 months; HR 0.42 (95% CI 0.26 to 0.68); p<0.001, figure 2B). The estimated OS at 1 year was 73.0% (95% CI 66.7% to 79.8%) vs 40.6% (95% CI 27.2% to 60.8%) in patients with an ECOG PS of 0–1 vs 2, respectively.

In total, 147 (62.8%) patients had confirmed or presumed disease progression on first-line pembrolizumab: 119 (61%) in the ECOG PS 0–1 group and 28 (71.7%) in the ECOG PS 2 group. Among patients who progressed on pembrolizumab and were alive, patients with an ECOG PS of 0–1 were significantly more likely to receive second-line systemic therapy compared with the ECOG PS 2 group (65% (65 of 100 patients) vs 22.2% (4 of 18 patients), p=0.001).

### Univariate and multivariate analyses

In addition to ECOG PS, other variables including PD-L1 expression levels, smoking status, and brain metastases (BM) also had signal of association with benefit to first-line pembrolizumab (defined as p<0.2 for either PFS or OS) in a univariate analysis (table 2), and were therefore included in a multivariate model (table 3). We also observed an interaction between ECOG PS and BM in the multivariate model for both PFS and OS. In multivariate analysis adjusting for PD-L1 level and smoking status, patients with ECOG PS 0–1 had significantly improved PFS (HR 0.54 (0.33 to 0.89); p=0.02) and OS (HR 0.29 (0.16 to 0.50); p<0.001) compared with ECOG PS 2 in patients without BM before the start of immunotherapy. Among patients with BM prior to initiation of first-line pembrolizumab, ECOG PS was no longer associated with PFS (HR 1.31 (0.62 to 2.73)) or OS (HR 1.06 (0.43 to 2.61)) (online supplementary figure 1).

10.1136/jitc-2020-001007.supp1 Supplementary data

**Table 2:** Univariate analyses of clinical factors for PFS and OS

	PFS		OS	
	HR (95% CI)	Log-rank p value	HR (95% CI)	Log-rank p value
**ECOG PS** 0–1 vs 2	0.70 (0.47 to 1.06)	0.09	0.42 (0.26 to 0.68)	**<0.001**
**Age** (<70 vs ≥70)	1.07 (0.78 to 1.49)	0.67	0.74 (0.50 to 1.12)	0.15
**Sex** Female vs male	1.16 (0.84 to 1.61)	0.36	1.20 (0.80 to 1.80)	0.39
**Smoking status** Current/ever vs never	0.62 (0.36 to 1.07)	0.08	0.87 (0.44 to 1.73)	0.70
**Histology** Non-squamous vs squamous	0.78 (0.50 to 1.22)	0.27	0.86 (0.49 to 1.49)	0.59
**KRAS mutation** No vs yes	0.85 (0.61 to 1.20)	0.36	0.78 (0.51 to 1.19)	0.25
**Brain metastases** Present vs absent	1.28 (0.90 to 1.83)	0.17	1.24 (0.80 to 1.92)	0.34
**PD-L1 expression** 90%–100% vs 50%–89%	0.65 (0.47 to 0.90)	**0.01**	0.58 (0.38 to 0.87)	**0.01**

ECOG PS, Eastern Cooperative Oncology Group Performance Status; OS, overall survival; PD-L1, programmed cell death ligand 1; PFS, progression-free survival.

**Table 3:** Multivariate analyses of clinical factors for PFS and OS

		PFS		OS	
		HR (95% CI)	P value	HR (95% CI)	P value
**ECOG** **PS** 0–1 vs 2	**BM** Present	1.31 (0.62 to 2.73)	0.48	1.06 (0.43 to 2.61)	0.93
	**BM** Absent	0.54 (0.33 to 0.89)	**0.02**	0.29 (0.16 to 0.50)	**<0.001**
**PD-L1 expression** 90%–100% vs 50%–89%		0.67 (0.48 to 0.93)	**0.02**	0.59 (0.39 to 0.89)	**0.01**
**Smoking status** Ever vs never		0.74 (0.43 to 1.28)	0.28	1.04 (0.52 to 2.10)	0.90

BM, brain metastases; ECOG PS, Eastern Cooperative Oncology Group Performance Status; PD-L1, programmed cell death ligand 1.

## Discussion

Deciding on whether and how to treat patients with newly diagnosed advanced NSCLC and a poor performance status remains challenging in clinical practice. Because an impaired performance status might either be secondary to the underlying cancer disease burden, or related to other medical conditions and comorbidities, the risks and benefits of any systemic therapy must be considered carefully.^9^ With first-line cytotoxic chemotherapy, a poor performance status is associated with shortened survival and with the development of more severe toxicities compared with patients with a good PS.^10^ Despite this, NSCLC randomized trials have demonstrated a significant benefit for using platinum doublet chemotherapy over single-agent regimens with regard to both survival and quality of life in this population.^11 12^

Little is known about the efficacy of first-line immunotherapy-containing regimens for NSCLC in patients with a poor performance status since this population was excluded from all the seminal phase III clinical trials.^1–4 13 14^ Although the PePS2 study specifically evaluated the safety and tolerability of pembrolizumab in 60 patients with an ECOG PS of 2, only a small subset of 10 patients received pembrolizumab in the first-line setting with a PD-L1 TPS of ≥50%.^15^ Here, were report outcomes among 234 patients treated with first-line commercial pembrolizumab. Because non-clinical trial populations generally do not perform as well as clinical trial participants,^16^ we compared clinical outcomes in 39 patients with an ECOG PS of 2 to 195 patients with an ECOG PS of 0–1; all patients were treated outside the setting of a clinical trial to control for the potential impact of studying outcomes in a non-clinical trial group. Even though we observed a modest difference in mPFS between the ECOG PS 0–1 and 2 groups (6.6 months vs 4.0 months), we found a marked difference in mOS (20.3 months vs 7.4 months), respectively. This shortened OS in the ECOG PS 2 population likely reflects a further deterioration in performance status, precluding use of subsequent therapy, and only a fraction of patients in the ECOG PS 2 group received any second-line systemic therapy.

Although outcomes to first-line pembrolizumab in our cohort of patients with an ECOG PS of 2 were generally poor, we did observe a response rate of 25.6% in this population. While this efficacy may be comparable with the reported response rate of 24% to platinum doublet chemotherapy in the poor performance status population,^11^ single-agent pembrolizumab generally has a more favorable side effect profile than cytotoxic chemotherapy.^1^ Moreover, a considerable proportion (28.3%) of the ECOG PS 2 group remains on pembrolizumab without progression at the time of data analysis in our study. Therefore, there may be a subset of patients with a poor performance status who still derive significant and durable benefits with first-line immunotherapy. Further data from larger populations will be necessary to determine which patients are most likely to experience prolonged disease control despite having a poor performance status.

Despite the favorable impact of ECOG PS 0–1 on clinical outcomes among patients without BM, we observed less of an impact of ECOG PS on outcomes among patients with BM. However, the number of patients with BM in each ECOG PS group was relatively small in our study and additional studies are needed to determine the impact of performance status on outcomes to immunotherapy in various subgroups of patients. Tools such as the diagnosis-specific graded prognostic assessment scale, which takes into account patient age, number of brain lesions, presence of extracranial metastases, and ECOG PS,^17^ may be more informative to gain a better understanding of clinical outcomes to immunotherapy in patients with BM.

There are several limitations to our retrospective study. First, determining a patient’s performance status is subjective and can vary among clinicians^5^ as well as between physicians and patients, as patients tend to rate themselves as having a worse ECOG PS than physicians ascribe to them.^5 16^ Furthermore, an important issue which this study did not assess is whether the use of immunotherapy in patients with a poor performance status is as safe and tolerable as in patients with a good ECOG PS. However, the CheckMate 153 and 171 trials of nivolumab in previously treated NSCLC showed that treatment was mostly safe in the ECOG PS 2 subgroup, with no apparent increase in the risk of immune-related or other toxicities compared with patients with a better ECOG PS.^6 7^ Nonetheless, our analysis represents the largest retrospective cohort of patients with advanced NSCLC and a PD-L1 TPS ≥50% with an impaired performance status treated with first-line pembrolizumab to date.

Although a proportion of patients in our study appear to have a prolonged benefit to first-line pembrolizumab, survival in this patient population remains short. Prospective studies will hopefully help define the best initial treatment approach for this vulnerable population. Preliminary data from CheckMate 817 of first-line ipilimumab plus nivolumab in patients with either an ECOG PS 2 or with a comorbidity (such as asymptomatic untreated brain metastases, hepatic or renal impairment, HIV)^18^ showed a promising mPFS of 9.6 months in patients with PD-L1 TPS ≥50% (n=33). Another prospective study (eNERGY, NCT03351361) will compare first-line ipilimumab plus nivolumab with carboplatin-based doublet chemotherapy in patients with an ECOG PS 2. Given the large numbers of patients who have an impaired performance status at the time of their initial lung cancer diagnosis, additional strategies will be necessary to determine the optimal treatment paradigms for this population.
