# Supplementary material for: Outcomes to first-line pembrolizumab in patients with PD-L1-high (≥50%) non–small cell lung cancer and a poor performance status
Source: J Immunother Cancer. 2020 Aug 4;8(2):e001007. doi: 10.1136/jitc-2020-001007 (PMC7406027; doi:10.1136/jitc-2020-001007)
Supplement: Supplementary data [file jitc-2020-001007supp001.pdf]

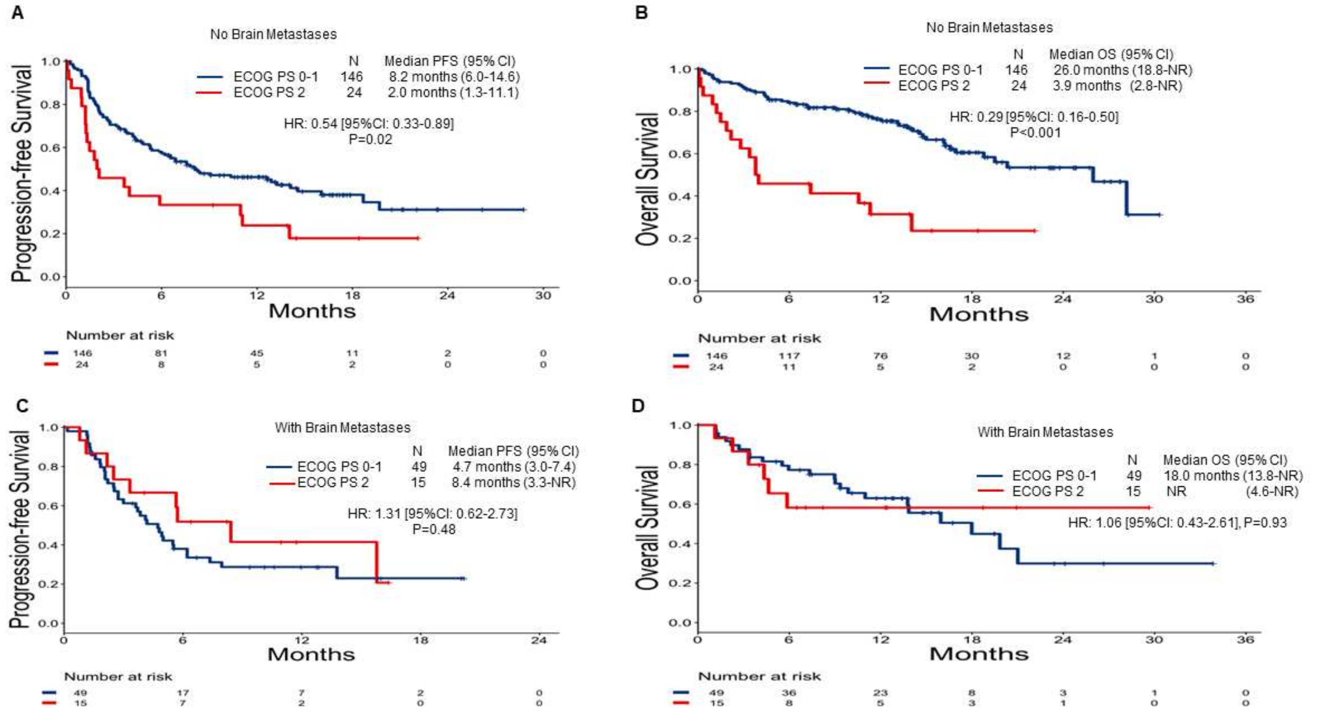

Supplementary Figure 1 Kaplan-Meier estimates by ECOG PS groups for patients without brain metastases (**A**, progression-free survival; **B**, overall survival), and in patients with a history of brain metastases (**C**, progression-free survival; **D**, overall survival).
